# A novel PIBF1-RET gene fusion identified from a stage IA lung adenocarcinoma: A case report

**DOI:** 10.1097/MD.0000000000034305

**Published:** 2023-07-21

**Authors:** Weidi Zhao, Jia’en Sun, Huangkai Zhu, Guofang Zhao

**Affiliations:** a School of Medicine, Ningbo University, Ningbo, China; b Department of Thoracic Surgery, Hwa Mei Hospital, University of Chinese Academy of Sciences, Ningbo, China.

**Keywords:** case report, non-small cell lung cancer (NSCLC), novel fusion partner, PIBF1, resectable, RET

## Abstract

Rearranged during transfection (RET) gene fusions occur in 0.7% to 2% in lung cancer and 1% to 2% in non-small cell lung cancer. Systemic therapies for RET fusion-positive non-small cell lung cancer consist mostly of targeted therapy with RET inhibitors such as selpercatinib and pralsetinib. To date, approximately 40 fusion partners have been reported. Herein, we report a novel progesterone immunomodulatory binding factor 1 (PIBF1)-RET gene fusion identified from a stage IA lung adenocarcinoma and was further validated by RNA sequencing analysis.

**Patient concerns:**

A 55-year-old male smoker was found by chest computed tomography to have a solid nodule in the right lower lobe of the lung and enlarged mediastinal lymph nodes.

**Diagnoses:**

The patient was then diagnosed with stage IA lung adenocarcinoma (T1N0M0).

**Intervention:**

The patient then underwent thoracoscopic lobectomy of the right lower lobe and mediastinal lymph node dissection. Molecular testing with a targeted panel of 8 lung cancer-associated driver genes detected a novel PIBF1-RET (P16:R12) fusion, which putatively encodes a gene in which the first 16 exons of PIBF1 was concatenated to RET exon 13 and its downstream sequence, retaining the RET kinase domain. The genomic translocation was further validated by RNA sequencing with a panel of 115 cancer-associated genes, which found no other aberrations.

**Outcomes:**

The patient was discharged 3 days after surgery.

**Conclusion:**

We report a novel PIBF1-RET fusion in early-stage lung adenocarcinoma. This finding expands the spectrum of RET fusion partners and warrants further studies in characterizing the oncogenic role of this genomic aberration and response to RET-targeted therapies.

## 1. Introduction

Rearranged during transfection (RET) gene fusions occur in 0.7% to 2% in lung cancer and 1% to 2% in non-small cell lung cancer (NSCLC).^[1,2]^ Located on chromosome 10q11.2, RET encodes a transmembrane tyrosine kinase that could activate pivotal tumor-promoting signaling pathways such as PI3K/Akt and rat sarcoma/mitogen-activated protein kinase. In-frame RET gene fusions that retain the kinase domains encoded by exons 13 to 16 could lead to ligand-independent, constitutive activation of RET kinase activity, which could drive tumor initiation and growth.^[3]^ Systemic therapies for RET fusion-positive NSCLC consist mostly of targeted therapy with RET inhibitors such as selpercatinib^[4]^ and pralsetinib,^[5]^ both recently approved as first-line treatment. Approximately 40 fusion partners have been reported to date according to the Atlas of Genetics and Cytogenetics in Oncology and Haematology (https://atlasgeneticsoncology.org/gene/76/ret-(rearranged-during-transfection).^[6]^ A large-scale characterization of Chinese lung cancer revealed a small number of highly prevalent partners, including kinesin family member 5B, coiled coil domain containing 6, and nuclear receptor coactivator 4, which collectively accounted for ~75% of all RET fusions identified in the study.^[2]^ Nonetheless, daily clinical practice continues to uncover new partners. Herein, we report a novel progesterone immunomodulatory binding factor 1 (PIBF1)-RET gene fusion identified from a stage IA lung adenocarcinoma and was further validated by RNA sequencing analysis.

## 2. Case presentation

**Figure 1.**
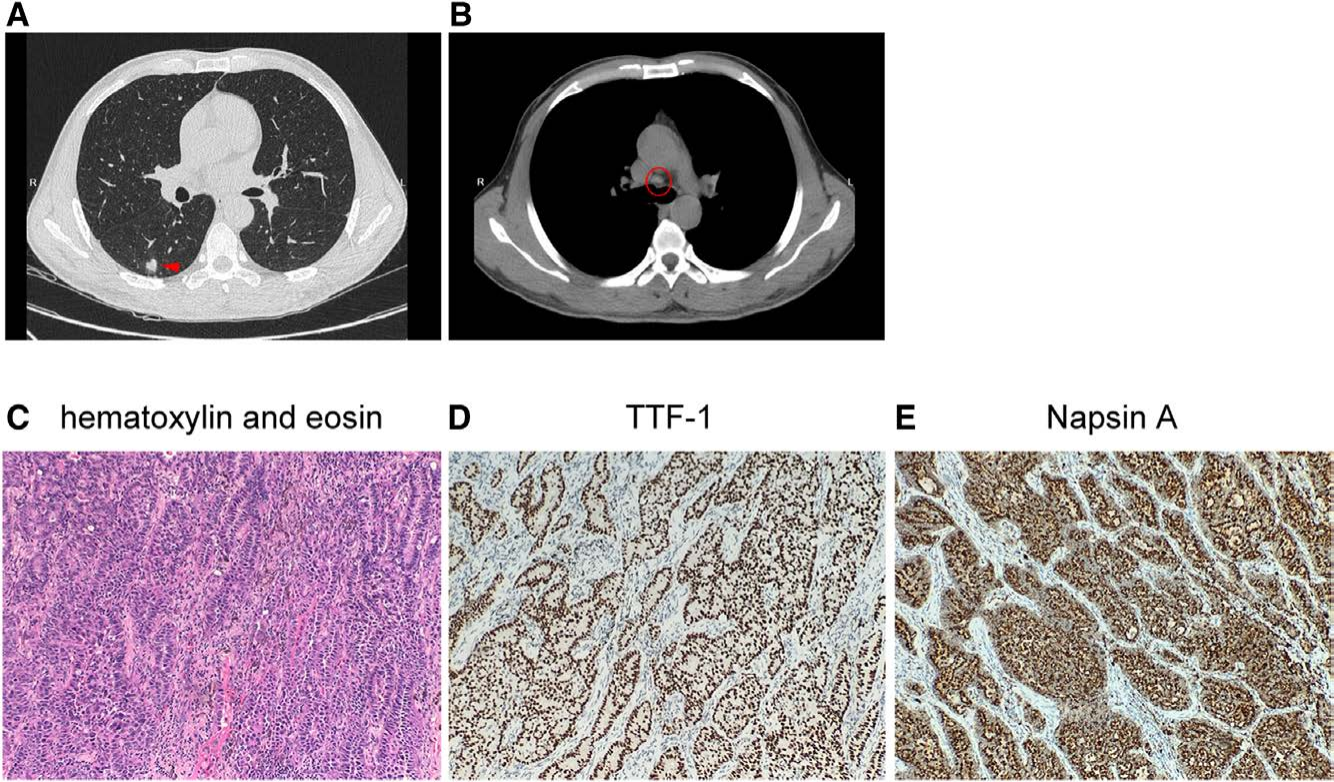
Imaging and histopathologic findings of the described case. Chest computed tomography detected (A) a nodule in the right lower lobe (arrowhead) and (B) slightly enlarged mediastinal lymph node (circle). (C) Hematoxylin and eosin staining of the resected tumor tissue, which stained positively for (D) TTF-1 and (E) napsin A on immunohistochemistry. All micrographs are under 100× magnification. TTF-1 = thyroid transcription factor-1.

**Figure 2.**
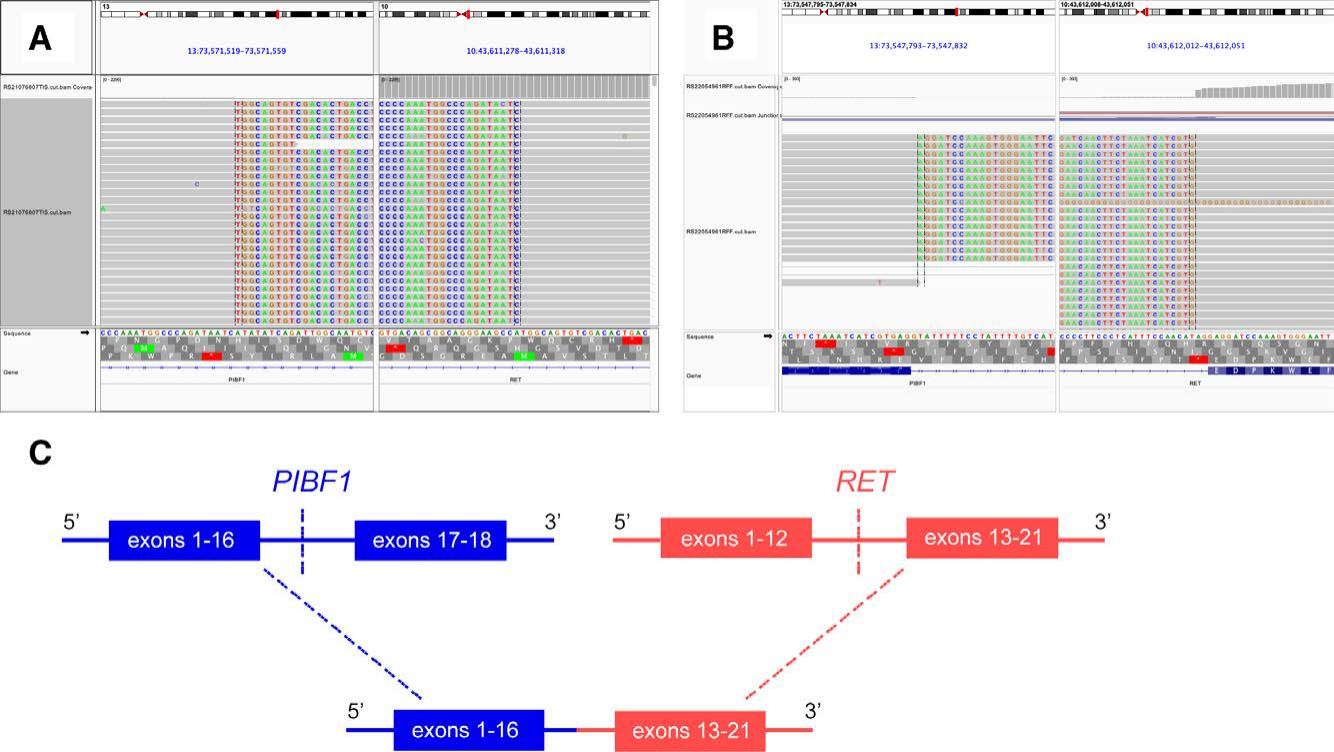
Structure of the novel PIBF1-RET gene fusion identified from a resected lung adenocarcinoma. Integrative Genomics View snapshots showing the structure identified in (A) DNA and (B) RNA sequencing. (C) A schematic illustration showing the formation of the fusion gene. PIBF1 = progesterone immunomodulatory binding factor 1, RET = rearranged during transfection.

A 55-year-old male smoker of 30 pack-years visited our center after detection by chest computed tomography of a mass in the right lower lobe (RLL) of the lung during a physical checkup 2 weeks earlier. Past medical history was unremarkable. There was no family history of cancer. The patient was asymptomatic. Physical examination noted no palpable lymph nodes, subcutaneous emphysema, or chest pain upon pressing. Chest percussion note was resonant, and tactile fremitus and breath sounds were normal with no rales or pleural friction rubs. He was therefore scheduled for further examinations after 3 weeks of anti-inflammation treatment. Upon follow-up in May 2022, chest computed tomography detected a solid nodule in RLL and slightly enlarged mediastinal lymph nodes (Fig. 1A and B). Brain magnetic resonance imaging was unremarkable. The patient then underwent thoracoscopic lobectomy of the RLL and dissection of mediastinal lymph nodes. Histopathologic review of the resected tumor (1.1 cm in greatest dimension) revealed an invasive adenocarcinoma that stained positively for TTF-1 (+), napsin A (+) and negatively for CK5/6, P40, and P63. No nodal metastasis was noted in the lymph node specimens (Fig. 1C–E). The patient was therefore diagnosed with stage IA (T1N0M0) NSCLC. Molecular testing with a targeted panel of 8 lung cancer-associated driver genes (Burning Rock Biotech, Guangzhou, China) detected a novel PIBF1-RET (P16:R12) fusion (abundance 10.3%), which putatively encodes a gene in which the first 16 exons of PIBF1 was concatenated to RET exon 13 and its downstream sequence, retaining the RET kinase domain (Fig. 2A and B). The genomic translocation was further validated by RNA sequencing analysis of the resection (OncoRNA, Burning Rock Biotech, Guangzhou, China; Fig. 2C). The patient was discharged 3 days later and scheduled for regular follow-up visits.

## 3. Discussion

To our knowledge, this is the first report describing the genomic rearrangement between PIBF1 and RET in NSCLC. Both DNA and RNA sequencing of the resected tumor suggested the presence of intact RET kinase domain in the resulting chimeric protein. No other genomic aberration was detected by targeted DNA (with an 8-gene panel) or RNA (115-gene panel) sequencing analyses. These 2 findings suggested the PIBF1-RET fusion as the oncogenic driver in this case, although more preclinical and clinical validation is still warranted. In terms of targeted therapy options, the chimeric protein may be susceptible to inhibition by RET inhibition by selpercatinib or pralsetinib, although in this case no adjuvant treatment was administered to this stage IA (T1N0M0) patient. More evidence is therefore needed to better characterize the PIBF1-RET fusion. In conclusion, we provide clinical evidence of a novel PIBF1-RET oncogenic fusion in early-stage lung adenocarcinoma. This finding expands the spectrum of RET fusion partners and warrants further studies in characterizing the oncogenic role of this genomic aberration and response to RET-targeted therapies.

## Acknowledgments

We would like to thank the patient and her family for their support. We are also grateful to Xiao Zou, Wenjie Sun, Jiaqi Chu, and Jinlei Song from Burning Rock Biotech for technical assistance.

## Author contributions

**Conceptualization:** Weidi Zhao, Jia’en Sun, Huangkai Zhu, Guofang Zhao.

**Data curation:** Weidi Zhao, Jia’en Sun, Huangkai Zhu, Guofang Zhao.

**Formal analysis:** Weidi Zhao, Jia’en Sun, Huangkai Zhu, Guofang Zhao.

**Funding acquisition:** Guofang Zhao.

**Supervision:** Guofang Zhao.

**Writing – original draft:** Weidi Zhao, Jia’en Sun, Huangkai Zhu, Guofang Zhao.

**Writing – review & editing:** Weidi Zhao, Jia’en Sun, Huangkai Zhu, Guofang Zhao.
